# Very Late‐Onset Myasthenia Gravis Presenting With Dysphagia and Gradual Decrease in Laryngeal Elevation During Repeated Swallowing: A Case Report

**DOI:** 10.1002/ccr3.70434

**Published:** 2025-04-17

**Authors:** Yujuan Han, Dawei Zang, Xiaoping Kang

**Affiliations:** ^1^ Tiantan Xiaotangshan Rehabilitation Center Beijing Xiaotangshan Hospital Beijing China; ^2^ Department of Rehabilitation Medicine Beijing Tiantan Hospital, Capital Medical University Beijing China

**Keywords:** dysphagia, elderly, myasthenic crisis, upper gastrointestinal radiography, very late‐onset myasthenia gravis

## Abstract

Very late‐onset myasthenia gravis (VLOMG) is rare myasthenia gravis (MG) that begins after the age of 65 years. Here, we describe a 72‐year‐old patient who presented with dysphagia. Upper gastrointestinal radiography revealed delayed initiation of swallowing in the pharynx and inadequate opening of the upper esophageal sphincter. Notably, there was a gradual decrease in laryngeal elevation, which strongly suggested MG. Additionally, the frequency of swallowing was reduced, and anti‐acetylcholine receptor (anti‐AChR) antibodies were positive. Due to type I respiratory failure, the patient required a tracheotomy and ventilator support. By the time of hospital discharge, the patient was able to walk out independently with a stomach tube and tracheotomy cannula. In conclusion, VLOMG should be considered in patients over 65 years old who present with sudden, isolated dysphagia or dysarthria, particularly when there is a gradual decrease in laryngeal elevation during swallowing as observed on upper gastrointestinal radiography.

**Summary**

- For patients over 65 years old who present with sudden, isolated dysphagia, particularly when upper gastrointestinal radiography reveals a progressive decline in laryngeal elevation during repeated swallowing, myasthenia gravis, very late‐onset myasthenia gravis in particular, should be considered.

## 1 Introduction

Myasthenia gravis (MG) is an autoimmune disease mediated by acetylcholine receptor antibodies [1], and very late‐onset MG (VLOMG) is a rare condition defined as MG that begins after the age of 65 [2, 3, 4]. Recent epidemiological studies have shown a gradual increase in the incidence of MG among elderly patients [5, 6]. MG‐related mortality peaks in both adolescent males and the elderly in China [6]. Patients with VLOMG often have other underlying conditions that complicate diagnosis and treatment. To date, only a few studies have focused on VLOMG [3, 7, 8].

Here, we present a case of a 72‐year‐old patient with VLOMG who exhibited sudden dysphagia as the primary symptom. Notably, during upper gastrointestinal radiography, there was a gradual decrease in laryngeal elevation while swallowing repeatedly.

## 2 Case Presentation

A 72‐year‐old male patient presented with dysphagia that had persisted for 1.5 months. He was admitted to our department on August 6, 2023, and discharged on December 6, 2023, after a total of 4 months of treatment. The patient had a history of lung cancer, which was resected 6 years prior, and Parkinson's disease that was diagnosed 3 years ago. Prior to the onset of his current symptoms, the manifestations of Parkinson's disease, including bradykinesia and resting tremors, had significantly improved, although he continued to experience a mild festinating gait. Following the onset of dysphagia, he lost approximately 10 kg over the course of 1.5 months.

## 3 Differential Diagnosis, Investigations, and Treatment

On physical examination, the patient was in fair general condition with moderate wasting but was not anemic. He underwent a comprehensive assessment to evaluate deglutition function, motor function, activities of daily living, and neurological function. The assessment revealed a weakened pharyngeal reflex and dysphagia, as indicated by the water swallowing test. Although limb movement was normal, the patient experienced balance disorders. The Barthel Index score was 80, the Berg Balance Scale score was 38, and the Mini‐Mental State Examination score was 28 (with a high school education level).

**FIGURE 1:**
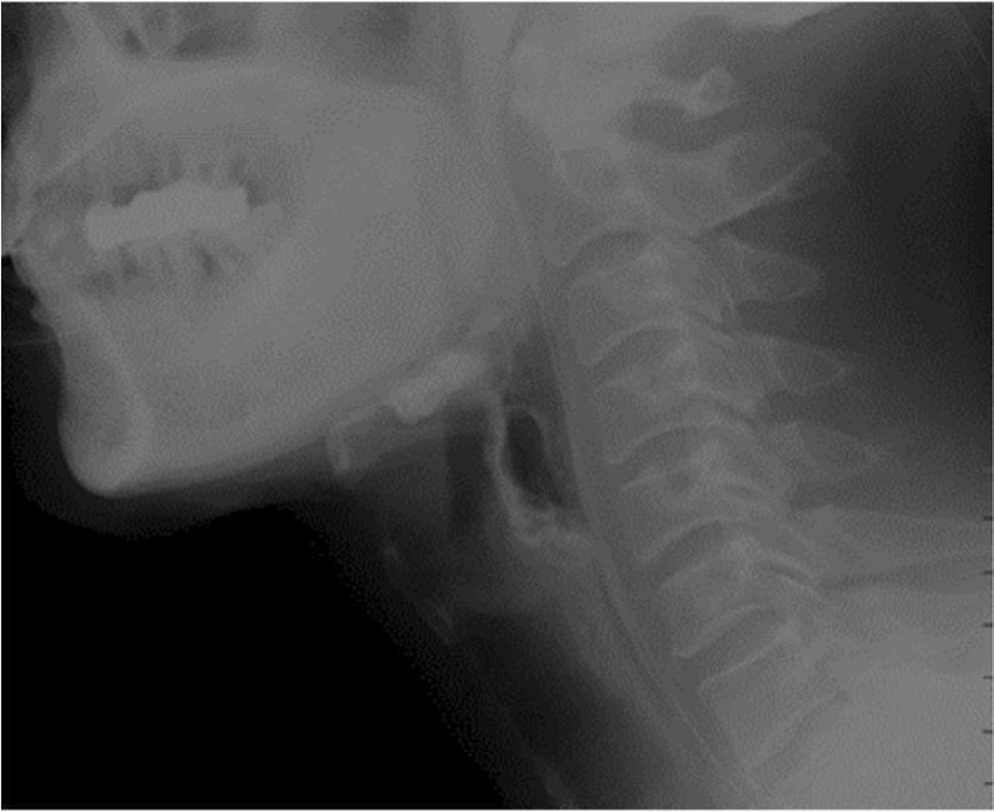
Insufficient laryngeal elevation and upper esophageal sphincter opening during swallowing.

**FIGURE 2:**
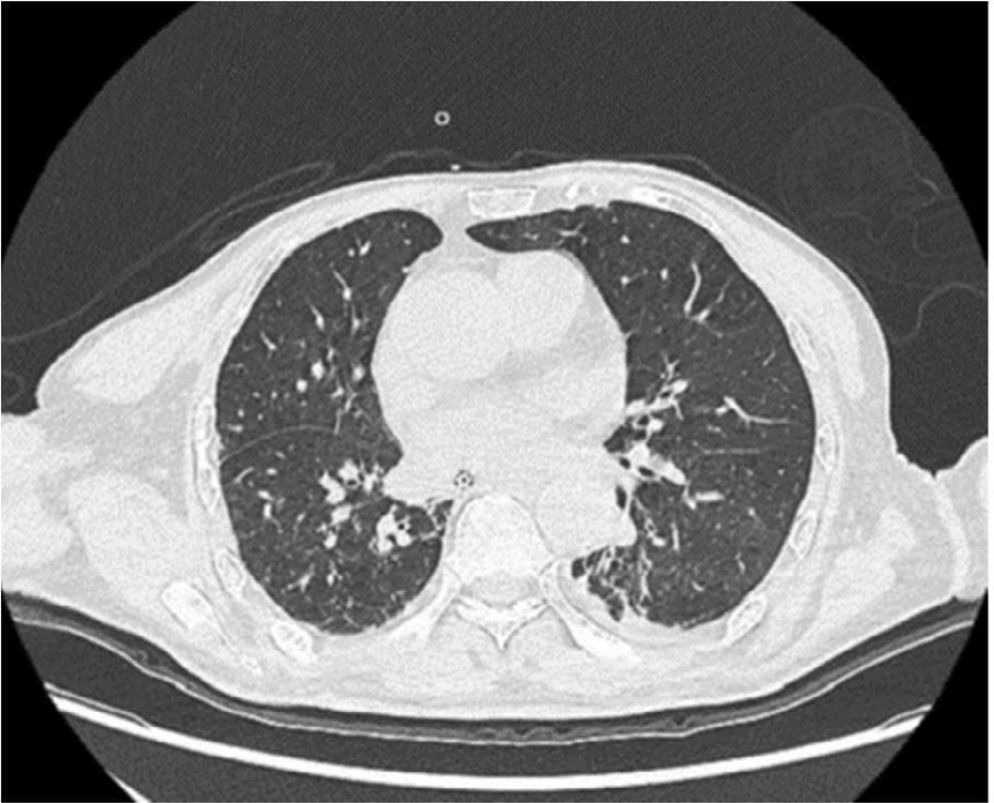
Lung CT showed bronchiectasis and bilateral pneumonia.

Upper gastrointestinal radiography demonstrated decreased mobility of the tongue base, delayed initiation of swallowing in the pharynx, and insufficient laryngeal elevation and upper esophageal sphincter opening. Notably, laryngeal elevation gradually decreased during repeated swallowing (Figure 1). Lung CT revealed right upper lobe atelectasis with bronchiectasis and bilateral pneumonia (Figure 2). Based on these findings, MG could not be excluded, and therefore, an electromyography (EMG) examination was planned.

To ensure adequate nutrition, an indwelling nasogastric tube was placed. Systematic rehabilitation included swallowing, balance, and walking therapies. After 1 week of treatment, the patient showed significant improvement in walking and balance, but swallowing difficulties persisted. Before EMG could be performed, the patient was transferred to the intensive care unit (ICU) due to sudden respiratory failure, which was likely related to MG. Notably, the patient had experienced a mild upper respiratory tract infection 4 days prior to the onset of respiratory failure.

In the ICU, a tracheotomy was performed, and the patient received ventilator‐assisted breathing. Repetitive electrical nerve stimulation tests conducted in the ICU indicated decreased low‐frequency responses when stimulating the axillary and facial nerves. Additionally, anti‐acetylcholine receptor (anti‐AChR) antibodies were positive (> 8.01 nmol/L, with a positive threshold > 0.50 nmol/L). These findings confirmed the diagnosis of MG. A follow‐up lung CT scan showed no thymoma, ruling out secondary MG. Given the patient's age of onset (72 years), the diagnosis was VLOMG.

Given the patient's respiratory failure and after excluding respiratory and central nervous system diseases based on clinical and imaging assessments, a myasthenic crisis was diagnosed. Symptomatic treatments, including pyridostigmine bromide and intravenous immunoglobulin, were administered. The patient received two courses of intravenous immunoglobulin therapy, each lasting 5 days with 500 mL administered daily, separated by a 3‐week interval. Pyridostigmine bromide was given orally at a dose of 60 mg four times per day. Additionally, anti‐infective and other supportive treatments were provided.

## 4 Outcome and Follow‐Up

Following treatment, the patient's condition gradually stabilized. After leaving the ICU, the patient did not receive aggressive immunosuppressive or hormonal therapy because of the complex medical history related to lung cancer. This condition likely contributed to the lack of significant improvement in swallowing function.

Upon discharge, the patient still had an indwelling stomach tube and tracheotomy cannula. The water swallowing test continued to indicate dysphagia, and upper gastrointestinal radiography showed no significant changes compared to previous examinations. However, the Berg Balance Scale score improved to 43, and the Barthel Index score was 75. At 6 months of follow‐up, the patient successfully removed the stomach tube and tracheotomy cannula. His activities were nearly normal, and he was able to travel with his family. The Barthel Index score improved to 95, and the Berg Balance Scale score reached 49.

## 5 Discussion

VLOMG is a rare condition defined as the onset of MG after the age of 65 [1]. The atypical presentation of VLOMG can make diagnosis challenging. Patients may exhibit symptoms such as drooping eyelids, eye movement disorders, apathy, dysphonia, and dysphagia. These symptoms often need to be differentiated from cerebrovascular disease and motor neuron disease, which can complicate the timely detection and diagnosis of VLOMG [10]. Diagnostic laboratory tests include repeated nerve stimulation (e.g., a decrement of ≥ 10% in the amplitude of the compound muscle action potential between the first and the fourth or fifth stimulation at low‐frequency stimulation, according to EMG), neostigmine tests, and acetylcholine receptor antibody tests. In China, thymoma is commonly associated with late‐onset MG (LOMG) [11], and the positive rate of anti‐AChR antibodies is higher in VLOMG patients compared to those with early‐onset MG and LOMG (92.9%) [2]. Additionally, bulbar symptoms such as dysphagia tend to appear earlier in VLOMG patients. In our case, we promptly conducted EMG, acetylcholine receptor tests, and thymoma screening as soon as the patient's condition allowed. These diagnostic measures were instrumental in making a timely and accurate diagnosis.

The typical clinical manifestation of MG is fluctuating skeletal muscle fatigue or weakness. However, in elderly patients with neurological diseases such as cerebrovascular disease and Parkinson's disease, acute neurological symptoms—such as extraocular muscle weakness, dysphagia, dysarthria, and limb weakness—may be easily overlooked or misdiagnosed. Previous studies suggest that MG should be considered in elderly patients presenting with sudden and isolated dysarthria or dysphagia [11, 12]. One study reported that swallowing dysfunction occurs in 41.7% of patients with late‐onset MG [10]. Our patient, a 72‐year‐old with a history of Parkinson's disease and lung cancer, presented with dysphagia. Upper gastrointestinal radiography revealed delayed pharyngeal swallowing, inadequate laryngeal elevation, and insufficient upper esophageal sphincter opening, with a gradual decrease in laryngeal elevation during repeated swallowing. Additionally, repetitive nerve electrical stimulation showed decreased low‐frequency responses, and anti‐AChR antibodies were positive—findings consistent with the majority of existing literature [13, 14]. Thus, for atypical cases of VLOMG, timely performance of repetitive nerve stimulation, anti‐AChR antibody testing, and upper gastrointestinal radiography is crucial for accurate diagnosis and treatment.

In this case, a myasthenic crisis occurred during hospitalization, primarily presenting as sudden Type I respiratory failure. Myasthenic crisis is one of the most severe complications of MG and can be life‐threatening. Neumann et al. [16] have shown that the incidence of myasthenic crisis in elderly patients with MG is increasing, often due to infections. Other studies have reported that approximately 15%–20% of MG patients develop a myasthenic crisis within the first 2–3 years after diagnosis and may require invasive or noninvasive ventilation [17]. In our case, the myasthenic crisis developed about 2 months after symptom onset, amidst atypical symptoms and a complex medical history. The likely trigger was an upper respiratory tract infection. Four days after the onset of sudden respiratory failure, the patient was admitted to the ICU and underwent tracheostomy with ventilator‐assisted ventilation. After active rescue and symptomatic treatment, the patient's condition gradually stabilized. Although the patient was able to breathe spontaneously, tracheal intubation was maintained postweaning to ensure continued respiratory support. The patient had a history of Parkinson's disease for more than 3 years and a history of lung cancer for more than 6 years. Parkinson's disease or Parkinson's syndrome can also be the first or main manifestation of dysphagia. Schiffer et al. [18] examined 68 patients with Parkinson's disease who underwent upper gastrointestinal angiogram; the results suggested that the airway closure was slower during swallowing and the hyoid bone was raised and relaxed. This is different from the characteristics observed in dysphagia caused by MG and can be used for differentiation.

Paraneoplastic syndromes associated with lung cancer can also lead to dysphagia, often due to esophageal achalasia linked with malignant tumors [19]. To distinguish dysphagia caused by paraneoplastic syndrome, clinicians should be vigilant for other clinical manifestations of these syndromes. This patient developed dysphagia 6 years after the diagnosis and treatment of lung cancer. Notably, the relief and improvement of symptoms occurred independently of any antitumor interventions, making a paraneoplastic syndrome unlikely in this case.

Once a clear diagnosis of VLOMG was established, our patient received prompt treatment, including pyridostigmine bromide, immunoglobulin, anti‐infective agents, and other supportive therapies. Due to the complex medical history related to lung cancer, the patient did not receive aggressive immunosuppressive or hormonal therapy. At discharge, the patient still had an indwelling stomach tube and tracheotomy cannula. Despite this, significant improvements were observed in balance function and daily living abilities. After 6 months of follow‐up, the patient successfully removed the stomach tube and tracheotomy cannula and returned to near‐normal activities. Joy Vijayan et al. also reported that patients with LOMG generally have a good prognosis and treatment response. Comorbidities such as cardiorespiratory, musculoskeletal, or neurological disorders did not significantly impact the severity of myasthenic symptoms or outcomes in these patients [20]. Our findings are consistent with this literature.

In summary, for patients over 65 years old who present with sudden and isolated dysphagia, particularly if there is a gradual decrease in laryngeal elevation during repeated swallowing as seen on upper gastrointestinal radiography, MG (especially VLOMG) should be considered. Timely diagnostic evaluations and interventions are crucial to prevent infections and other complications, ensuring early detection, accurate diagnosis, and appropriate treatment.

## Author Contributions

**Yujuan Han:** conceptualization, formal analysis, investigation, methodology, software, visualization, writing – original draft. **Dawei Zang:** data curation, resources, supervision, validation, writing – review and editing. **Xiaoping Kang:** conceptualization, methodology, project administration, supervision, writing – review and editing.

## Ethics Statement

Informed written consent was obtained from the patient before writing this case report.

## Consent

Written informed consent was obtained from the patient.

## Conflicts of Interest

The authors declare no conflicts of interest.

## Data Availability

The datasets used and/or analyzed during the current study are available from the corresponding author upon reasonable request.
